# Architecture and molecular machinery of skeletal myofibers: a systematic review of the structure–function relationships

**DOI:** 10.3389/fcell.2025.1602607

**Published:** 2025-05-20

**Authors:** Jing Zhao, Xu Du, Wei Fang, Hongxia Zhu, Binglin Yue

**Affiliations:** ^1^ Department of Endocrinology, Chengdu Shuangliu Hospital of Traditional Chinese Medicine, Chengdu, China; ^2^ Key Laboratory of Qinghai-Tibetan Plateau Animal Genetic Resource Reservation and Utilization, Sichuan Province and Ministry of Education, Southwest Minzu University, Chengdu, China

**Keywords:** skeletal myofiber, myofibril, sarcoplasmic reticulum, mitochondria, cytoskeleton

## Abstract

The skeletal muscle, one of the largest tissues in mammals, plays a crucial role in maintaining body movement and energy metabolism. Dysfunction or damage to the skeletal muscle can lead to various muscle diseases, such as muscular dystrophy, myasthenia, and others. The myofiber presents the fundamental structural unit of the skeletal muscle, and research on its structure and biological function is of great significance. Here, we review the latest progress in the structural and functional aspects of the myofiber, focusing on myofibril, the sarcoplasmic reticulum, mitochondria, and the cytoskeleton. The basic properties and dynamic interactions of a large number of muscle proteins have been described in detail, including the scaffold construction of core protein components and the fine-tuning of secondary protein components with functional redundancy. This overview provides new insights into skeletal muscle pathophysiology.

## 1 Introduction

The skeletal muscle, the most abundant tissue in vertebrates (accounting for ∼40% of total body weight), is primarily composed of myofibers with minor contributions from adipose, vascular, nervous, and connective tissues. Recent advances in single-cell sequencing, however, have unveiled its intricate cellular diversity, including multinucleated myofibers, muscle stem cells, endothelial cells, immune cells, adipocytes, neurocytes, and other mononuclear cell populations (Cai et al., 2023a). This cellular heterogeneity not only defines the tissue architecture but also enables efficient communication strategies between cell types to facilitate the exchange of biological information, thereby maintaining skeletal muscle homeostasis (Cai et al., 2023b; Krauss et al., 2017). Beyond its structural complexity, the skeletal muscle plays indispensable roles in basic mammalian body functions such as locomotion, respiration, and metabolism. These functions depend on a tightly orchestrated developmental program: in vertebrates, skeletal muscle ontogeny requires successive phases of fetal, postnatal, and adult myogenesis (Salvatore et al., 2014; Greggio et al., 2017; Egan and Zierath, 2013; Relaix et al., 2021). Central to this process are the main waves of myogenesis during *in utero* development, when myogenic progenitor cells located on the peripheral edge of the dermomyotome extend downward to form the myotome, which rapidly differentiates into spindle mononuclear myoblasts (Deries and Thorsteinsdottir, 2016). Following migration to muscle formation sites, these myoblasts undergo proliferation, differentiation, and fusion to assemble multinucleated, contractile myofibers (Buckingham et al., 2003). Postnatally, skeletal muscle growth shifts focus: myofiber numbers remain largely constant, with growth instead driven by hypertrophy and the regenerative activity of satellite cells in response to damage (Yin et al., 2013). Underpinning these developmental and regenerative processes is a well-established transcriptional hierarchy including master regulators such as Pax3, Pax7, myogenic regulatory factors (MRFs), and the myocyte enhancer factor 2 (MEF2) family (Bryson-Richardson and Currie, 2008; Asfour et al., 2018). While this transcriptional framework is well-characterized, the structural and functional complexity of myofibers—particularly their subcellular components—remains incompletely understood. To address this gap, we review key myofiber structures such as myofibrils, the sarcoplasmic reticulum (SR), mitochondria, and the cytoskeleton, focusing on their molecular architecture and functional mechanisms to advance our understanding of skeletal myogenesis.

## 2 Structure and composition of the skeletal myofiber

Skeletal muscle functionality hinges on the specialized organization of myofibers, which are multinucleated syncytia unique to terrestrial animals. These elongated cells, spanning centimeters in length and 10–100 µm in diameter, are surrounded by connective tissues (endomysium, perimysium, and epimysium) that stabilize the muscle architecture and transmit mechanical forces (Powell et al., 2002). However, the functional essence of myofibers resides in their internal components: myofibrils, dominating the cytoplasmic space; these tightly packed bundles of actin and myosin filaments form repeating sarcomeres—the contractile units responsible for muscle shortening; SR, a specialized endoplasmic reticulum enveloping myofibrils; the SR stores and releases calcium ions to regulate excitation–contraction coupling; mitochondria: positioned near the sarcolemma and between myofibrils; mitochondria generate ATP through oxidative phosphorylation, sustaining energy-demanding contractions; cytoskeleton: composed of costameres, intermediate filament (IF), and microtubules; this network maintains sarcomere alignment, distributes mechanical stress, and anchors organelles such as the nuclei and mitochondria. These components collaborate dynamically: myofibrils drive contraction, the SR synchronizes calcium signaling, mitochondria fuel activity, and the cytoskeleton integrates structural integrity.

Myofibers also harbor specialized structures critical to their function: the neuromuscular junctions (NMJs) for nerve signal transmission; myotendinous junctions (MTJs) for force transmission to tendons; and cilia, which have recently been implicated in mechanosensory signaling (Brooks et al., 2023; Ng et al., 2021). Notably, myofiber size adapts dynamically through radial hypertrophy (increase in diameter) or longitudinal growth (addition of sarcomeres in series), although the molecular drivers of these processes remain incompletely characterized. Surrounding the myofibers is a hierarchical network of connective tissues. From the innermost to the outermost, these include the following: endomysium, a thin layer of collagen and proteoglycans enveloping individual myofibers; perimysium, a thicker sheath encircling bundles of myofibers (fascicles), rich in blood vessels and nerves; and epimysium, a dense collagenous membrane encasing the entire muscle. These layers serve dual structural and functional roles: they transmit mechanical forces during contraction, anchor NMJs and MTJs, and provide a scaffold for satellite cell-mediated regeneration (Cretoiu et al., 2018). Intriguingly, recent studies suggest that connective tissues—not myofibers themselves—bear the majority of mechanical loads during muscle activity (Gillies and Lieber, 2011). Connective tissues externally reinforce this system, but the molecular interplay within myofibers ultimately dictates muscle performance and adaptability.

### 2.1 Myofibril

**FIGURE 1:**
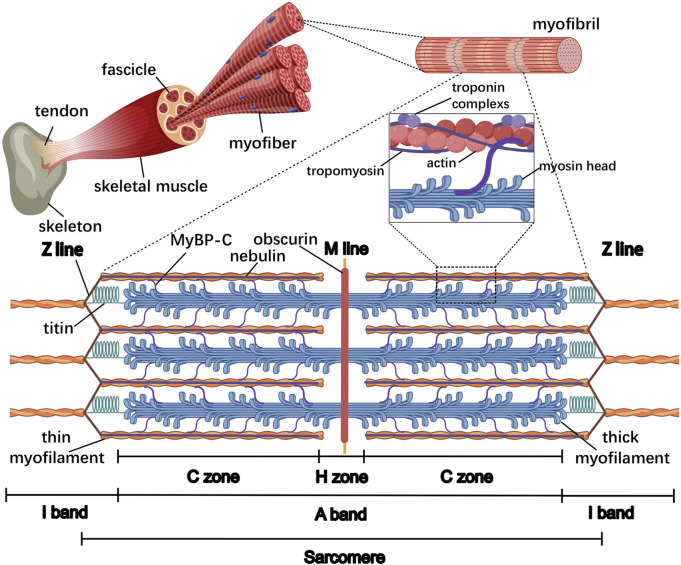
General organization of the sarcomere in the skeletal muscle. From the inside to the outside, the skeletal muscle comprises the myofiber, fascicle, and whole muscle, respectively. Within the myofiber, the myofibrils are aligned along the longitudinal axis of the cell body and almost occupy the entire intracellular volume, and the sarcomere is the smallest contractile unit of the myofiber. The major lines and bands are indicated, as well as myofilament-associated proteins such as thin and thick myofilaments, MyBP-C, titin, nebulin, and obscurin. The thin and thick myofilaments overlap at the C zone in the A-band where 7–9 MyBP-C stripes connect with both myofilaments; titin is directed toward its N-terminus at the Z-line and its C-terminus at the M-line; nebulin is aligned with its C terminus in the Z-line; obscurin is oriented with its N-terminus in the M-line and its C-terminus bound to the SR.

It is easily observable under the electron microscope that myofibers filled with myofibrils are aligned along the longitudinal axis of the cell body. The myofibril has a diameter of approximately 1 μm and presents a periodic striped structure alternating between light and dark, in which, each I-band (light area) is divided into two parts by a Z-line and each A-band (dark area) is divided into two portions by an M-line within the H zone. The segment of myofibrils between two adjacent Z lines is commonly described as a sarcomere, which is the smallest contractile unit of the striated muscle (Figure 1). On the meta-microscale, the sarcomere primarily consists of thin and thick myofilaments that are perpendicular to the Z-line: the actin, tropomyosin, and troponin complexes (troponin T, I, and C, respectively) are organized into thin myofilaments that are anchored in Z-lines, while the myosins are assembled into thick myofilaments in the A-band that overlap with thin myofilaments (Ertbjerg and Puolanne, 2017). These structures form the basis for the “sliding filament theory” of skeletal muscle contraction: muscle contraction entails the thin myofilaments sliding over the thick myofilaments into the A-band, causing the shortening of the I-band and H zone, while the widths of the Z-line, A-band, and M-line remain unchanged (Huxley and Niedergerke, 1954; Huxley and Hanson, 1954). Specifically, myosin heads bind actin at a ∼45° angle, forming the acto-myosin crossbridges, which pull actin filaments toward the M-line through ATP hydrolysis-driven conformational changes, resulting in sarcomere shortening. Additionally, approximately 30% of crossbridges maintain passive muscle tension even during relaxation, mediated by the spring-like domains of structural titin in coordination with residual crossbridge interactions to preserve myofiber elasticity (Al-Khayat, 2013). In addition, other myofilament-associated proteins such as myosin binding protein-C (MyBP-C), nebulin, and obscurin have also been proposed to play important structural and regulatory functions during the assembly of sarcomeres.

For instance, MyBP-C is a myofilament protein binding to thin and thick myofilaments maintaining the ordered arrangement of the sarcomere and cross-bridge cycling (van Dijk et al., 2014). The interaction of MyBP-C with both myofilaments is restricted to the C zone, where actin and myosin overlap in the A-band, and MyBP-C is arranged in 7–9 transverse stripes at 43-nm intervals (Luther and Craig, 2011). MyBP-C consists of a series of immunoglobin (Ig) and fibronectin III (Fn)-like domains, and the MyBP-C N-terminal binds to actin and myosin head, while the MyBP-C C-terminal binds with meromyosin and titin (Squire et al., 2003). In addition, accumulating evidence has implicated MyBP-C in the regulation of myofilament Ca^2+^ sensitivity and enzymatic activity of myosin (van Dijk et al., 2014).

As the largest known polypeptide, titin spans the length of half of the sarcomere, anchoring its N-terminus and C-terminal at the Z-line and M-line, respectively. The C-terminals of two titin molecules overlap on both sides of the M-line, forming a continuous titin filament harboring tight binding sites for a variety of sarcomeric proteins, which functions as a scaffold, mechanical sensor, and signaling mediator. For example, within the Z-line, titin N-terminus interacts with proteins such as α-actinin and telethonin to maintain the structural integrity and resist high mechanical stress (Young et al., 1998; Mues et al., 1998). Similarly, titin C-terminal binds with several proteins at the M-line, including Muscle RING finger-1 (MURF-1), myomesin, and M-protein, functioning as scaffolding structures for A-band assembly (Witt et al., 2005). Titin is normally regarded as a “molecular spring” that generates passive tension, and the elements responsible for this elasticity primarily reside in the I-band region of titin, including tandemly arranged Ig-domains, PEVK motifs, and N2A element (Linke et al., 1998; Linke et al., 2002; Granzier and Labeit, 2006; Granzier and Labeit, 2005). Unlike the elastic I band region of titin, the A‐band region of titin is inextensible and provides a scaffold for myosin and MyBP-C, therefore determining the length and structure of thick myofilaments (Whiting et al., 1989). Moreover, as its I-band and M-line region contain Ser/Thr kinase domains, titin may be involved in signaling transduction from myofibrils to other compartments of myofiber, contributing to muscle adaptation in conditions of mechanical stress changes (Tskhovrebova and Trinick, 2008; Puchner et al., 2008).

Ultrastructural results with monoclonal antibodies have established that nebulin is oriented with its N-terminus at the tips of thin myofilaments in the I-band and its C-terminus at the Z-line, whose configuration both stabilizes the thin myofilaments and regulates the Z-line structure (Wright et al., 1993; Wang and Wright, 1988; Witt et al., 2006). Actually, nebulin is non-extensible because nebulin epitopes maintain a fixed distance from the Z-line, regardless of sarcomere stretching (Kruger et al., 1991). Numerous studies indicate that nebulin can bind to various proteins, including actin and myosin components, as well as proteins associated with its unique N- and C-terminal regions, especially for the central super repeats of nebulin, through which nebulin laterally interacts with the thin myofilament proteins actin, tropomyosin, and troponin complexes, functioning as the scaffold to control thin myofilament length (Kruger et al., 1991; Labeit et al., 1991). In addition, nebulin’s super-repeat region also binds to myosin, which may inhibit actomyosin ATPase activity and the sliding velocity of actin on myosin in a calcium-/calmodulin-dependent manner (Root and Wang, 1994). Differing in sequence from the central super repeats, the N-terminal sequence carries glutamate residues and can interact with tropomodulin to specify thin myofilament length, whereas the C-terminus is rich in serine residues, and the SH3 domain can directly bind to Z-line protein myopalladin and CapZ and titin to mediate the myofibrillar assembly and mechanochemical signaling (Mcelhinny et al., 2001; Wear et al., 2003; Bang et al., 2001; Kontrogianni-Konstantopoulos et al., 2009).

In contrast to MyBP-C, titin, and nebulin presenting within sarcomeres, obscurin wraps around myofilaments over the M-line and Z-line, contributing to their assembly and integration with other sarcoplasmic elements such as the SR (Kontrogianni-Konstantopoulos et al., 2003). Given its structure composing of tandem adhesion modules and signaling domains, obscurin can associate tightly and periodically with the myofilaments and the periphery of the myofibril. Specifically, the N-terminal Ig domain of obscurin binds directly to myofibrillar components such as titin, myomesin, and MyBP-C, while obscurin tightly connects with the SR component small ankyrin-1 (sAnk1) through the C-terminus to its last Ig-like domain (Kontrogianni-Konstantopoulos and Bloch, 2005; Porter et al., 2005). Other kinetic studies showed that in addition to structural support function, obscurin has a possible involvement in RhoA- and Ca^2+^-mediated signaling pathways via its Rho-GEF/PH and IQ motif, and the presence of obscurin at the NMJ is consistent with this, suggesting its direct or indirect involvement in signal transduction at the synaptic terminal (Fukuzawa et al., 2005; Young et al., 2001; Carlsson et al., 2008). Initial subcellular distribution of obscurin studies using antibodies indicated that obscurin briefly accumulates at the Z-line and primarily concentrates at the M-line during embryogenesis; however, it eventually loses Z-line distribution in later developmental stages (Young et al., 2001). Actually, multiple alternatively spliced forms of obscurin exist in distinct sarcomeric locations, such as obscurin A, obscurin B, and several additional variants that differ in the combination of C-terminal structural domain elements and localize to the M-line, A/I junction, and Z-line at the muscle resting state, respectively (Bowman et al., 2007). Notably though, when muscles are stretched, obscurin can redistribute to different locations along the sarcomere length (Bowman et al., 2007). Considering the preferential integration of these obscurin isoforms with certain regions of the sarcomere, it further implies specialized functions of these isoforms during myofibrillogenesis and at maturity. Collectively, myofibrillogenesis depends on the coordinated assembly and integration of a large number of myofilament-associated proteins into the sarcomeres. These molecules may interact with several protein ligands to function as scaffolds, regulate signaling cascades, and localize them to particular sites within or surrounding sarcomeres to coordinate sarcomeric arrangement.

### 2.2 Sarcoplasmic reticulum

**FIGURE 2:**
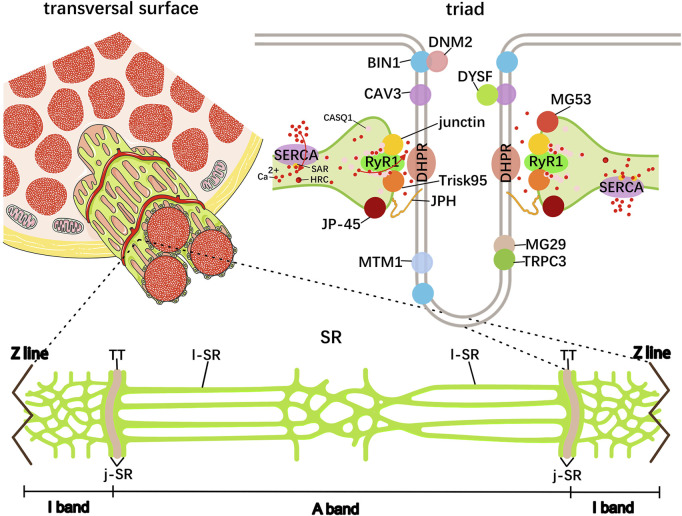
Schematic representation of skeletal muscle SR. The elaborated SR network consists of tubules and cisternae around each myofibril shown in green. The central TT and the terminal cisternae element on both sides collectively form the triad structure, in which many protein–protein interactions are responsible for Ca^2+^ cycling and storage. These proteins include dihydropyridine receptor (DHPR), ryanodine receptor 1 (RyR1), TT biogenesis proteins amphiphysin 2/bridging integrator-1 (BIN1), caveolin 3 (CAV3), and dysferlin (DYSF); triad formation proteins mitsugumins (MG), transient receptor potential cation channel type 3 (TRPC3), junctophilin (JPH), myotubularin (MTM1), triadin, junctin, and the junctional sarcoplasmic reticulum protein 1 (JP-45); and Ca^2+^-binding proteins calsequestrin (CASQ), sarcalumenin (SAR), and HRC.

The skeletal muscle contains a specialized endoplasmic reticulum network called SR, which is responsible for protein and calcium homeostasis regulation. Highly precise views under electron microscopes (EMs) showed that the SR consists of tubules and cisternae around each myofibril, in which the thin tubules are named longitudinal SR (l-SR) and are positioned around the A- and I-band, and its enriched Ca^2+^ ATPase (SERCA) is responsible for removing Ca^2+^ from the cytoplasm to the lumen; the tubule edges merge into the terminal cisternae at the boundaries of the A- and I-band and collectively form the triad structure, with the transverse tubules (TT) originating from the sarcolemma, maintaining excitation–contraction coupling (ECC) in the skeletal muscle (Rossi et al., 2022a) (Figure 2; Table 1). Within the muscle field, ECC is defined as the process linking the sarcolemma depolarization induced by motor–neuron stimulation and Ca^2+^ release from the SR. In particular, there are two Ca^2+^ channel proteins dihydropyridine receptor (DHPR) and ryanodine receptor 1 (RyR1) located on the TT and terminal cisternae region facing the TT terminal junctional SR (j-SR), respectively. Following sarcolemma depolarization, the DHPR undergoes conformational changes and directly transmits signals to open the RyR1 to trigger Ca^2+^ release into the myoplasm, thereby initiating muscle contraction (Barone et al., 2015). In fact, DHPR and RyR1 tightly wound together to form the core of the macromolecular complex known as the Ca^2+^ release unit, which includes numerous interacting proteins, and each of them influences the entire ECC process (Dulhunty, 2006). These proteins include TT biogenesis proteins amphiphysin 2/bridging integrator-1 (BIN1), caveolin 3 (CAV3), and dysferlin (DYSF); triad formation proteins mitsugumins (MG), junctophilin (JPH), myotubularin (MTM1), triadin, junctin, and the junctional sarcoplasmic reticulum protein 1 (JP-45); and Ca^2+^-binding proteins calsequestrin (CASQ), sarcalumenin (SAR), and HRC (Figure 2). For instance, BIN1 is highly expressed in the skeletal muscle and capable of driving membrane tubulation during TT maturation, in which BIN1 works separately or in cooperation with a GTPase dynamin 2 (DNM2) (Lee et al., 2002; Cowling et al., 2017). BIN1 knockout mouse myofibers present abnormal TTs and deficient ECC, and DNM2 downregulation improved its motor and histopathological phenotypes, suggesting that the balance between BIN1 and DNM2 is necessary for TT maturation, which is mediated by the interaction of the BIN1 SH3 domain with the proline-rich domain of DNM2 (Silva-Rojas et al., 2022; Fujise et al., 2021; Takei et al., 1999). As a member of the integral membrane protein family, CAV3 is not only implicated in caveolae membrane formation at the sarcolemma but also localizes at the developing TTs, and its deletion leads to TT membrane disorder with lateral loss. However, CAV3 is undetectable in the mature TTs, suggesting that CAV3 may be involved in the early development of the TT system in the skeletal muscle (Tang et al., 1996; Parton et al., 1997; Galbiati et al., 2001). DYSF mainly localizes to the sarcolemma for membrane fusion and repair, and DYSF-deficient muscle shows subsarcolemmal vacuoles contiguous with the TT system (Selcen et al., 2001). Similarly to mice deficient in CAV3, DYSF mouse mutants display dilated and longitudinally oriented TTs, and CAV3 and DYSF show partial colocalization and interaction in developing TT system, suggesting a specific role of DYSF in TT biogenesis (Hernandez-Deviez et al., 2006; Matsuda et al., 2001). Although the precise mechanism is not yet clear, there is a hypothesis that DYSF may facilitate the fusion of vesicles containing CAV3 with TTs (Klinge et al., 2010). These findings suggest that TT biogenesis requires advanced mechanisms of membrane fusion, and additional factors involved in TT formation are needed.

**TABLE 1:** Summary of key muscle proteins and their roles.

Protein	Localization	Primary function	Key interactions	References
MyBP-C	C zone of the A-band	Maintains the ordered arrangement of the sarcomere and cross-bridge cycling and modulates myofilament Ca^2+^ sensitivity and myosin enzymatic activity	Myosin, titin, and actin	(van Dijk et al., 2014; Luther and Craig, 2011; Squire et al., 2003)
Titin	Z-disk to M-band	Functions as a scaffold, mechanical sensor, and signaling mediator	α-actinin, telethonin, and MyBP-C	(Young et al., 1998; Mues et al., 1998; Witt et al., 2005, Linke et al., 1998, Linke et al., 2002, Granzier and Labeit, 2006; Granzier and Labeit, 2005; Whiting et al., 1989; Tskhovrebova and Trinick, 2008; Puchner et al., 2008)
Nebulin	Z-disk to I-band	Controls thin myofilament length, actomyosin ATPase activity, myofibrillar assembly, and mechanochemical signaling	Actin, tropomyosin, troponin complexes, and myosin	(Wright et al., 1993; Wang and Wright, 1988; Witt et al., 2006; Kruger et al., 1991; Labeit et al., 1991; Root and Wang, 1994; Mcelhinny et al., 2001; Wear et al., 2003; Bang et al., 2001; Kontrogianni-Konstantopoulos et al., 2009)
Obscurin	M-line and Z-line	Integrates with other sarcoplasmic elements and directly or indirectly participates in signal transduction	Titin, myomesin, and MyBP-C	(Kontrogianni-Konstantopoulos et al., 2003; Kontrogianni-Konstantopoulos and Bloch, 2005; Porter et al., 2005; Fukuzawa et al., 2005; Young et al., 2001; Carlsson et al., 2008)
MG29	SR membrane and triad	Facilitates the docking and fusion of transport vesicles	TRPC3	(Nishi et al., 1999; Komazaki et al., 2001; Woo et al., 2015)
MG53	j-SR	Facilitates membrane repair at injury sites	CAV3 and DYSF	Cai et al. (2009b)
JPH	TT and j-SR	Provides a structural basis for physiological coupling between the plasma membrane and SR and serves as a scaffold for assembling Ca^2+^-release complexes to ensure effective ECC	DHPR, RyR, CAV3, and triadin	(Takeshima et al., 2000; Perni and Beam, 2022; Golini et al., 2011)
MTM1	TT	Mediates endocytosis and membrane trafficking	PtdIns3P and PtdIns(3,5)P2	Hnia et al. (2012)
Trisk 95	Terminal cisternae	Maintains the triad organization and intracellular Ca^2+^ homeostasis regulation	RyR1, JPH1, and CASQ1	(Shen et al., 2007; Rossi et al., 2022b; Lee et al., 2001)
Junctin	Terminal cisternae	Assures transmitting signals from CASQ1 to RyR1	RyR1 and CASQ1	(Dulhunty et al., 2009; Beard et al., 2008; Dulhunty et al., 2017)
JP-45	j-SR	Mediates voltage-dependent Ca^2+^-release	DHPR and CASQ1	(Anderson et al., 2003; Anderson et al., 2006; Gouadon et al., 2006; Yasuda et al., 2013)
CASQ1	SR	Stores 80% of the Ca^2+^ in the SR	RyR1, triadin, and junctin	(Beard et al., 2004; Murphy et al., 2009; Wang and Michalak, 2020; Royer and Rios, 2009)
SAR	SR	Enhances SERCA complex stability to pump Ca^2+^ into the lumen of the SR to initiate muscle relaxation	SERCA	(Yoshida et al., 2005; Conte et al., 2023)
HRC	SR	Manages Ca^2+^ release and uptake	RyR1 and SERCA	(Suk et al., 1999; Arvanitis et al., 2011)
DGC	Underneath the sarcolemma	Connects the cytoskeletal actin and the laminin	Dystrophin and DAGs	(Blake et al., 2002; Sweeney and Barton, 2000; Jaka et al., 2015)
Talin	Underneath the sarcolemma	Facilitates actin cytoskeleton interactions with the surrounding matrix	Integrin and vinculin	Mukund and Subramaniam (2020)
Desmin	Around the Z-line	Maintains cytoskeletal stability	Vimentin, nestin, synemin, paranemin, and syncoilin	(Capetanaki et al., 1997; Castanon et al., 2013)
Nestin	Around the Z-line, NMJ, and MTJ	Forms filaments	Vimentin and desmin	(Romero et al., 2013; Hol and Capetanaki, 2017; Cizkova et al., 2009)
Synemin	Around the Z-line	Links the heteropolymeric IFs to the sarcolemma	Desmin/vimentin, α-actinin, α-dystrobrevin, and vinculin	(Sun et al., 2008; Etienne-Manneville, 2018)
Lamin A/C	Beneath the inner nuclear membrane	Forms a nucleoskeleton and holds mechano signals	SUN1/2	Etienne-Manneville (2018)
Syncoilin	Near the sarcolemma, NMJ, and MTJ, and around the nuclei	Links desmin to the sarcolemma and contributes to the lateral force transmission	Desmin and α- dystrobrevin	(Moorwood, 2008; Blake and Martin-Rendon, 2002)

An abundance of membrane proteins are involved in triad formation, including the maturation of SR terminal cisternae and the connection between TT and SR. A notable example is the mitsugumin 29 (MG29), a member of the synaptophysin family that appears preferentially at the SR membrane and is then transferred to the triad proteins during the early stages of skeletal myogenesis (Takeshima et al., 1998; Komazaki et al., 1999). Analysis of MG29-deficient mice exhibited an accumulation of transport vesicles and incomplete formation of triad and SR networks, suggesting that MG29 may facilitate the docking and fusion of transport vesicles, processes that lead to the creation of the SR network and TT to form the triad (Nishi et al., 1999; Komazaki et al., 2001). Moreover, MG29-deficient mice showed impaired store-operated Ca^2+^ entry (SOCE) in the skeletal muscle, in which MG29 directly interacts with canonical-type transient receptor potential cation channel type 3 (TRPC3) on TT membranes to regulate Ca^2+^ transients during skeletal muscle contraction (Woo et al., 2015). Another synaptophysin, MG53 (also termed TRIM72), contributes to intracellular vesicle translocation and accumulation, and it has been recognized as an essential component of the membrane repair machinery in the striated muscle (Cai et al., 2009a; Whitson et al., 2021). It has been suggested that MG53 can interact with CAV3 and DYSF to facilitate membrane repair at injury sites by forming a molecular complex (Cai et al., 2009b).

In the skeletal muscle, JPHs (JPH1 and JPH2) provide a structural basis for physiological coupling between the plasma membrane and SR: they bridge the TT membrane through their cytosolic N-terminal domain with the j-SR via their C-terminal transmembrane domain (Takeshima et al., 2000). Apart from their structural functions, JPHs can also interact with various proteins such as DHPR, RyR, CAV3, triadin, and CASQ, serving as a scaffold for assembling Ca^2+^-release complexes to ensure effective ECC (Perni and Beam, 2022; Golini et al., 2011).

MTM1 is a phosphoinositide phosphatase localized at triads, and in phosphoinositide metabolism, MTM1 can specifically dephosphorylate phosphatidyl-inositol-3-phosphate (PtdIns3P) and phosphatidyl-inositol-3,5-bisphosphate (PtdIns(3,5) P2), which mediate endocytosis and membrane trafficking (Hnia et al., 2012). Moreover, MTM1 knockout in animal models leads to disruption of triad morphology and Ca^2+^ homeostasis, while its overexpression causes membrane stack accumulation in the subsarcolemmal region, indicating the delicate balanced expression of MTM1 in triad formation (Al-Qusairi et al., 2009; Dowling et al., 2009; Buj-Bello et al., 2008).

Triadin is an integral span membrane protein family localized at the triad, and all isoforms share common N-terminal transmembrane domains but have different C-terminal segments (Marty et al., 2009; Oddoux et al., 2009). The classic isoform of approximately 95 kDa (Trisk 95) is located at the terminal cisternae of the skeletal muscle, and its knockout mice model presented abnormal triads and Ca^2+^ homeostasis, but no significant changes were observed in ECC (Shen et al., 2007). It has been demonstrated that Trisk 95 is able to interact with RyR1, JPH1, and Ca^2+^-binding protein CASQ1 to maintain the triad organization and intracellular Ca^2+^ homeostasis regulation (Shen et al., 2007; Rossi et al., 2022b; Lee et al., 2001). Similarly, a membrane-spanning protein structurally homologous to triadin, named junctin, is an alternative splicing product of the aspartate β-hydroxylase gene (Dulhunty et al., 2009). Junctin and Trisk 95 both interact with RyR1 and CASQ1, anchoring CASQ1 near RyR1; however, their binding sites on RyR1 and CASQ1 and the detailed functional effects are extremely different. For example, in contrast to one same binding site (KEKE motifs) on Trisk 95 for RyR1 and CASQ1, there are multiple binding sites on junctin for RyR1 and CASQ1. Due to the presence of multiple RyR1/junctin and junctin/CASQ1 binding sites, compared to Trisk 95, junctin could assure transmittance of signals from CASQ1 to RyR1 (Beard et al., 2008; Dulhunty et al., 2017). Moreover, another integral membrane protein JP-45 is highly enriched in j-SR, which interacts with DHPR via its N-terminal domain and with CASQ1 through its C-terminal segment (Anderson et al., 2003). A substantial body of research indicates that both overexpression and ablation of JP-45 leads to a reduction in voltage-dependent Ca^2+^-release, which may be closely correlated with changes in the collaboration between JP-45 and DHPR/CASQ1 (Anderson et al., 2006; Gouadon et al., 2006; Yasuda et al., 2013).

It is well-known that Ca^2+^ is preserved in the SR and mainly buffered by the low-affinity and high-capacity Ca^2+^ binding protein CASQ to polymerize into elongated linear polymers under physiological conditions (Wang et al., 1998; Perni et al., 2013). Actually, CASQ is localized exclusively in the j-SR by binding to RyR, triadin, and junctin, as well as creating a complex three-dimensional cross-linked network. In mammals, the CASQ1 isoform is exclusively expressed in the fast-twitch skeletal muscle, while the CASQ2 isoform is found in the slow-twitch skeletal muscle and heart; especially, CASQ1 is capable of storing 80% of the Ca^2+^ in the SR (Beard et al., 2004; Murphy et al., 2009). It has become increasingly clearer that CASQ1 interacts with RyR, triadin, and junctin in a Ca^2+^-dependent manner, which is attributed to changes in the Ca^2+^ concentration that induces a conformational change in CASQ1: CASQ1 maximally inhibits the RyR1 when the free Ca^2+^ concentration reaches 1 mM, and the inhibition gradually diminishes as the Ca^2+^ concentration is altered, while CASQ1 dissociates from the RyR1 complex at Ca^2+^≤ 100 μM or≥ 5 mM (Wang and Michalak, 2020; Royer and Rios, 2009). Recently, new functions of CASQ1 have been proposed, including regulation of SOCE by binding to STIM1/OraiI and ER stress responses interacting with IRE1α in the skeletal muscle (Michelucci et al., 2020; Wang et al., 2019). Similar to the j-SR, the l-SR also contains Ca^2+^ binding proteins, and the most plentiful one is SAR. It has been demonstrated that SAR colocalizes and directly interacts with SERCA, enhancing SERCA complex stability to pump Ca^2+^ into the lumen of the SR to initiate muscle relaxation (Yoshida et al., 2005; Conte et al., 2023). Another secondary SR Ca^2+^-binding protein is HRC, which is far less abundant than CASQs but structurally similar to CASQs, functioning as Ca^2+^ buffer. Actually, the local and rapid changes of Ca^2+^ levels within the SR may result in different multimer forms of HRC and affect its interactions with other SR components (Suk et al., 1999). It has been suggested that HRC could interact with either the RyR or SERCA complex to manage Ca^2+^ release and uptake, respectively. As the SR Ca^2+^ increases, the HRC/SERCA interaction gradually diminishes, causing the detachment of SERCA from HRC. The released HRC then regulates Ca^2+^ release through the RyR complex, while locally reduced SR Ca^2+^ concentration facilitates the reconnection of HRC/SERCA, resulting in the reuptake of cytoplasmic Ca^2+^ (Arvanitis et al., 2011). However, current research studies on HRC mainly focus on the cardiac muscle, and the HRC/triadin and HRC/SERCA interactions and their functional implications on Ca^2+^-uptake and release in skeletal muscle remain unclear.

### 2.3 Mitochondria

As a highly metabolic tissue, the skeletal muscle relies on mitochondria to convert substrates into ATP via oxidative phosphorylation. This ATP powers critical processes such as myofibrillar contraction (through myosin ATPase activity), ion transport (e.g., Ca^2+^ reuptake by the SR), and cellular homeostasis. Beyond energy production, the mitochondria are central to metabolite biogenesis, cell-cycle regulation, and apoptosis/autophagy signaling (Mcbride et al., 2006; Dominy and Puigserver, 2013; Tilokani et al., 2018; Palmer et al., 2011; Ploumi et al., 2017). Actually, mitochondrial function is dynamically regulated by cycles of fission (division), fusion (merging), and motility—collectively termed mitochondrial dynamics (Ni et al., 2015). These processes depend on coordinated nuclear gene expression, mtDNA transcription, and protein import (Ploumi et al., 2017). Dysregulation of fission/fusion disrupts mitochondrial membrane integrity, exacerbating apoptosis and autophagy through pathways such as Bax/Bcl-2-imbalance and mPTP opening (Jeong and Seol, 2008; Xian and Liou, 2021).

The mitochondria in myofibers exhibit distinct morphological and functional specialization based on their subcellular localization: the sub-sarcolemmal mitochondria (SSM) reside beneath the sarcolemma and are usually spherical in form with few branches, supplying ATP to sarcolemmal pumps (e.g., Na^+^/K^+^-ATPase); the perinuclear mitochondria (PNM) are in proximity to nuclei/capillaries and have structural similarities with SSM, supplying ATP to the nuclear pores, and both SSM and PNM are termed peripheral mitochondria (PM); the intra-myofibrillar mitochondria (IFM) are located between myofibrils and have a close contact with the SR displaying reticular network structure, which specialize in ATP production for contractile machinery (myofibrils) and SR Ca^2+^-ATPase (SERCA) (Vincent et al., 2019; Swalsingh et al., 2022). IFM exhibit higher oxidative enzyme activity, respiratory chain complex density, and nucleotide metabolism proteins compared to SSM, aligning with their role in sustaining contraction (Ferreira et al., 2010).

Emerging studies demonstrate that mitochondrial subpopulations diverge in stress responses and signaling. For instance, some studies demonstrated that IFM possess higher levels of cytochrome c/apoptosis-inducing factor (AIF) release and lower membrane potential, ROS production rate, and Bax-to-Bcl-2 ratio compared with SSM (Adhihetty et al., 2005). Considering its greater oxidative enzyme activities and respiration rates, IFM may represent the larger pro-apoptotic protein pool in the skeletal muscle. A recent spatial distribution study of mitochondrial enzymes showed that complexes responsible for membrane potential (complex IV) and ATP (complex V) production are preferentially located in PM and IFM, respectively, and it has been postulated that membrane potential generated in PM can be directly distributed to IFM through mitochondrial connections, promoting ATP production (Willingham et al., 2021). It has also been reported that IFM is structurally and functionally associated with SR, and their contact sites are referred to mitochondria-associated membranes (MAMs) for exchanging Ca^2+^. In the mitochondria, Ca^2+^ influx increases ATP production by enhancing the activity of mitochondrial oxidases, while the exceeding physiological needs of Ca^2+^ can promote the opening of the mitochondrial permeability transition pore (mPTP), thereby triggering apoptosis or necrotic cell death (Rossi et al., 2019; Matuz-Mares et al., 2022). Therefore, mitochondrial subcellular specialization is tightly linked with skeletal muscle quality and function.

### 2.4 Cytoskeleton

**FIGURE 3:**
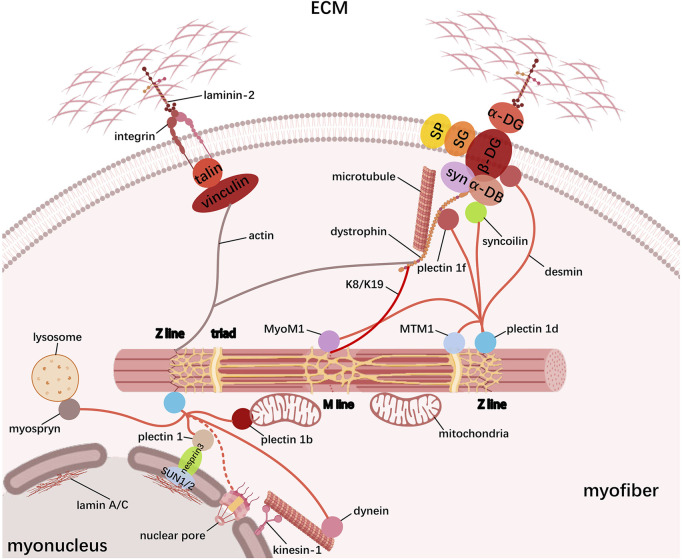
Schematic representation of the cytoskeleton in myofibers. Skeletal muscle cytoskeleton comprises myofibrils, costameres, IF, and microtubules. Costameres mainly contain the dystrophin–glycoprotein complex (DGC) and the vinculin–talin–integrin complex that bidirectionally link the ECM to myofibrils; IF proteins, especially desmin, tether the contractile myofibril to the sarcolemma and other organelles including myonucleus, mitochondria, SR, and lysosome, which forms the transverse fixation system of the muscle together with the costameres; microtubules form a grid containing longitudinal and transverse components across the myofiber to promote muscle maturation.

In the skeletal muscle, the cytoskeleton serves as a cellular scaffold determining the morphology of myofibers and transmitting mechanical force between myofibrils and extracellular skeleton. In addition to myofibrils themselves, the cytoskeleton component also includes costameres, IF, and microtubule. Costameres are positioned underneath the sarcolemma and vertical to the longitudinal axis of myofibers, connecting the sarcomere to sarcolemma via the Z-line and M-line. Along with assembling and stabilizing the sarcomere, costameres also regulate the interaction between the cytoskeleton and the extracellular matrix (ECM), all of which are based on its intricate protein composition. Current evidence supports the existence of major costameric proteins including the dystrophin–glycoprotein complex (DGC) and the vinculin–talin–integrin system (Figure 3). The DGC connects the cytoskeletal actin and the laminin via dystrophin and dystrophin-associated glycoprotein (DAG) interactions, respectively, in which the cytoskeletal protein dystrophin binds to cytoplasmic actin at its N-terminus, whereas its C-terminus is associated with DAGs that can be classified as cytoplasmic, supramembrane, and extracellular subcomplex (Blake et al., 2002; Sweeney and Barton, 2000). The cytoplasmic subcomplex comprises the α-dystrobrevin (α-DB) and syntrophin (syn), which are directly linked to dystrophin C-terminus. The supramembrane subcomplex includes glycosylated transmembrane protein sarcoglycan (SG) and sarcospan (SP), which need to be correctly assembled into a complex with dystrophin for maintaining sarcolemma integrity. For the extracellular subcomplex, the primary component α-dystroglycan (α-DG) strengthens the physical linkage between the ECM and the basement membrane components via binding to laminin-2 in the ECM and β-dystroglycan (β-DG) in the sarcolemma. The β-DG can also interact with the dystrophin, thus establishing the connection between the actin-based cytoskeleton and the ECM. Hence, it is clear that dystrophin serves a pivotal role in DGC system, whose absence or dysfunction leads to increased sarcolemma fragility, thereby weakening its functions to protect against mechanical stress (Jaka et al., 2015). Similarly, the vinculin–talin–integrin system is also fundamental for facilitating actin cytoskeleton interactions with the surrounding matrix, especially in skeletal muscle cell adhesion and signaling (Mukund and Subramaniam, 2020). Here, the dimeric protein talin links vinculin with integrin: on the one hand, talin binds to the cytoplasmic tails of integrins to activate ECM adhesion; on the other hand, the interaction between vinculin and talin provides the connection of actin filaments to the sarcolemma, reinforcing the adhesion site. Moreover, the complex can also participate in signal transduction pathways, which influence various cellular responses, such as changes in gene expression and cell behavior.

Another cytoskeletal element, IF, has been demonstrated to tether the contractile myofibrillar apparatus to the sarcolemma and other organelles such as the myonucleus, mitochondria, SR, and lysosome, which forms the transverse fixation system of the muscle together with the costameres (Figure 3). Based on similarities among their sequence and structure, IF proteins are grouped into six types, namely, types I and II (keratin), type III (desmin, vimentin, glial fibrillary acidic protein (GFAP), and peripherin), type IV (neurofilament-like protein), type V (lamin A/C), and type VI (large-molecular weight proteins such as nestin, synemin, paranemin, and syncoilin or a special group for CP49/phakinin and filensin/CP115), and the list is still growing (Hnia et al., 2015; Costa et al., 2004; Eldirany et al., 2021). During myogenesis, myoblasts initially express vimentin and nestin, while desmin is induced early in myogenic differentiation and then integrated into the pre-existing network of vimentin, and the mature skeletal muscle mainly contains desmin and keratin (Schweitzer et al., 2001). These filamentous structures form extended IF networks around the Z-line of the myofibrils, thereby linking adjacent myofibrils together, especially desmin and keratin (K8 and K19), which are linked to sarcolemma through costameric components. In addition, desmin knockout in mice causes disruption in the nuclear position, myofibril alignment, mitochondrial function, and even NMJ and MTJ integrity, indicating a more prominent role of desmin in maintaining cytoskeletal stability (Capetanaki et al., 1997; Castanon et al., 2013). Actually, molecular studies demonstrate that desmin can bind or copolymerize with IF proteins vimentin, nestin, synemin, paranemin, and syncoilin and widely interact with other functional proteins. In early myogenesis, nestin co-polymerizes with vimentin and later on with desmin to form filaments, whereas vimentin completely disappears and nestin is selectively expressed only in the NMJ and MTJ in mature skeletal myofibers (Romero et al., 2013; Hol and Capetanaki, 2017; Cizkova et al., 2009). Synemin is another desmin-associated molecule and is considered to link the heteropolymeric IFs to the sarcolemma by directly interacting with desmin/vimentin, α-actinin, α-dystrobrevin, and vinculin (a microfilament-associated protein) (Sun et al., 2008; Etienne-Manneville, 2018). For an IF component of developing skeletal muscle, paranemin has been shown to be required for desmin filaments organization, but not nestin or synemin (Schweitzer et al., 2001). Unlike the majority of IF proteins that form filaments by homopolymerization or heteropolymerization, syncoilin is a non-filament-forming member that binds directly to desmin and α-dystrobrevin, thus linking desmin to the sarcolemma and contributing to the lateral force transmission in the skeletal muscle. Except for being located near the sarcolemma, syncoilin is enriched at the NMJ and MTJ and around the nuclei in mature skeletal myofibers (Moorwood, 2008; Blake and Martin-Rendon, 2002). In the skeletal muscle, IFs are also located in the nuclei as lamins, mainly represented by lamin A/C, to form a nucleoskeleton beneath the inner nuclear membrane and hold mechanosignals. LINC complex (SUN1/2-nesprin3) on the nuclear membrane binds to lamin A/C at the inner nuclear membrane (through SUN1/2) and to desmin-binding protein plectin 1 at the outer nuclear membrane (through nesprin3), thus allowing the association of the nucleoskeleton with the cytoskeleton (Etienne-Manneville, 2018). Furthermore, via the nuclear pores, cytoplasmic IFs may directly connect lamins, but it remains to be determined in the skeletal muscle (Lockard and Bloom, 1993; Georgatos et al., 1987; Capetanaki et al., 2007). Desmin filaments have also been suggested to connect other cell components such as lysosome (via the myospryn), SR (via the MTM1), microtubule (via the dynein), and M-line (via the MyoM1) (Hnia et al., 2015), (Kielbasa et al., 2011). Especially, the desmin-binding protein plectins are responsible for linking desmin IF networks with nuclei (via the plectin 1), mitochondria (via the plectin 1b), Z-line (via the plectin 1 day), and costamere (via the plectin 1f) (Castanon et al., 2013), (Carlsson et al., 2000; Konieczny et al., 2008). These desmin interactions are likely to coordinate the activity of specific kinases and phosphatases, thus regulating signal transduction.

In addition to the costamere and IF, myofibers also contain microtubules to transmit mechanical force, which form a grid containing longitudinal and transverse components across the fiber. During myogenesis, myoblast fusion, nuclear localization to the cell periphery, and assembly of the sarcomere require microtubules to ensure proper myofiber structure and function (Lucas and Cooper, 2023). It is well known that microtubule assembly nucleates at the microtubule-organizing center (MTOC), and MTOC transferring from the centrosomal to non-centrosomal location is a marker of differentiation (Sanchez and Feldman, 2017). During skeletal muscle differentiation, mononuclear myoblasts cease proliferation and elongation to form myoblasts with fusogenic capability, attributed to the MTOC switching from centrosomes to nuclear membranes and bi-polar extension of microtubules toward the cell (Sanchez and Feldman, 2017; Clark et al., 2002). Myoblast fusion occurs at the tips of elongated myoblasts through close contacting and merging of the cell membranes; hence, the failure of myoblast elongation can lead to a lack of fusion (Kim et al., 2015). As fusion proceeds, the nucleus of a newly fused myoblast rapidly migrates to the central myotube nuclei, which is mediated by dynein motor–Par6 complex that connects the nuclear envelope with microtubules (Bone and Starr, 2016; Starr, 2017). Then, these nuclei spread apart and are evenly spaced throughout the myotube, and the process is currently thought to be regulated by a variety of mechanisms: the kinesin-1 motor-nesprin1/2 complex links the nuclei to the microtubules and transports the nuclei along the microtubules; the kinesin-1 motor-microtubule-associated protein 7 (MAP7) complex drives the sliding of anti-parallel microtubules that extend outward from adjacent nuclei, thereby pushing neighboring nuclei apart; Nesprin-α recruits centrosomal protein Akap450 to the nuclear envelope independently of kinesin to nucleate microtubules (Starr, 2017; Gimpel et al., 2017). Eventually, the nuclei move from the center of the myofiber to the sarcolemma in a desmin-dependent process; except for some nuclei that migrate to the NMJ and anchor there, the remaining nuclei are evenly spaced around the myofiber periphery (Bone and Starr, 2016). Moreover, microtubules bound by myosin-binding protein Muscle RING finger-2 (MURF-2) provide a scaffold for the association of sarcomeric myosin with the A-band portion of titin, thereby generating the mature A band during myofibril assembly (Pizon et al., 2002; Pizon et al., 2005). In a similar manner, another microtubule-binding protein dystrophin serves as the scaffold for microtubule growth, resulting in microtubules aligning with dystrophin in both longitudinal and transverse directions of the sarcomere (Prins et al., 2009). Currently, research on microtubule organization within myofibers is continually deepening, such as its involvement in fiber type-dependent, Golgi apparatus relevance, mitochondrial trafficking, and primary cilia signaling (Ng et al., 2021; Oddoux et al., 2013; Giovarelli et al., 2020).

## 3 Conclusion

Skeletal myogenesis is a lifelong process in mammals, governed by molecular mechanisms that remain both intricately orchestrated and incompletely resolved. While foundational regulators—including cyclin/CDK complexes, chromatin modifiers, and myogenic transcription factors—have been mapped, their spatiotemporal coordination during myofiber formation and adaptation is still emerging (Wu and Yue, 2024). Central to this process is the exquisite architectural organization of skeletal myofibers, where myofibrils, SR, mitochondria, and the cytoskeleton coalesce into a contractile machine. Myofibrils drive force generation, the SR and mitochondria synchronize excitation–energy coupling, and the cytoskeleton integrates mechanical and biochemical signals. This structural precision enables rapid, adaptable responses to physiological demands, but it also hinges on dynamic protein networks whose complexity we are only beginning to unravel.

In this review, we synthesized current knowledge on the structural and functional hierarchy of skeletal myofibers, emphasizing how core components (myofibrils, SR, mitochondria, and cytoskeleton) collaborate to enable contraction and homeostasis. Key findings include the following: myofibrils rely on scaffold proteins (e.g., titin and nebulin) for sarcomere assembly and minor regulators (e.g., α-actinin and telethonin) for stability under stress; SR–mitochondria crosstalk at mitochondria-associated membranes (MAMs) fine-tunes calcium signaling to balance ATP production and apoptosis; cytoskeletal networks (desmin and microtubules) distribute mechanical load and anchor organelles, linking contractile activity to nuclear reprogramming. Despite these advances, critical gaps persist. For example, the biological roles and molecular mechanisms of muscle protein components in skeletal myogenesis remain largely unknown, including what their signaling roles are within the myofiber architecture, what is the order of their interactions, how they are regulated and how they, in turn, regulate gene expression and homeostasis in myofibers; what mechanisms prioritize radial hypertrophy versus longitudinal growth during adaptation, and how do mechanical cues interface with transcriptional programs? Future progress will require leveraging emerging tools such as cryo-electron tomography to resolve subcellular structures *in situ*, single-cell multi-omics to deconvolve myonuclear specialization, and 3D biomimetic models to recapitulate niche-specific signaling. Building upon these methodological advances, further investigation of the nature of the dynamic and extraordinarily complicated interplay among muscle proteins using sophisticated molecular biology methods will have far-reaching implications on mammalian skeletal muscle pathophysiology.
